# Chemogenetic activation of endogenous arginine vasopressin exerts anorexigenic effects via central nesfatin-1/NucB2 pathway

**DOI:** 10.1186/s12576-021-00802-4

**Published:** 2021-06-16

**Authors:** Kenya Sanada, Mitsuhiro Yoshimura, Naofumi Ikeda, Kazuhiko Baba, Haruki Nishimura, Kazuaki Nishimura, Yuki Nonaka, Takashi Maruyama, Tetsu Miyamoto, Masatomo Mori, Becky Conway-Campbell, Stafford Lightman, Masaharu Kataoka, Yoichi Ueta

**Affiliations:** 1 Department of Physiology, School of Medicine, University of Occupational and Environmental Health, 1-1 Iseigaoka, Yahatanishi-ku, Kitakyushu, 807-8555 Japan; 2 Second Department of Internal Medicine, School of Medicine, University of Occupational and Environmental Health, Kitakyushu, 807-8555 Japan; 3 Department of Orthopaedic Surgery, School of Medicine, University of Occupational and Environmental Health, Kitakyushu, 807-8555 Japan; 4 Research Institute for Metabolism and Obesity, Maebashi, 371-0049 Japan; 5 Translational Health Sciences, Bristol Medical School, University of Bristol, Bristol, BS1 3NY UK

**Keywords:** Nesfatin-1/NucB2, Arginine vasopressin, Hypothalamus, Feeding, Transgenic rat, DREADDs

## Abstract

We examined whether the chemogenetic activation of endogenous arginine vasopressin (AVP) affects central nesfatin-1/NucB2 neurons, using a transgenic rat line that was previously generated. Saline (1 mL/kg) or clozapine-*N*-oxide (CNO, 1 mg/mL/kg), an agonist for hM3Dq, was subcutaneously administered in adult male AVP-hM3Dq-mCherry transgenic rats (300–370 g). Food and water intake were significantly suppressed after subcutaneous (s.c.) injection of CNO, with aberrant circadian rhythmicity. The percentages of Fos expression in nesfatin-1/NucB2-immunoreactive neurons were significantly increased in the hypothalamus and brainstem at 120 min after s.c. injection of CNO. Suppressed food intake that was induced by chemogenetic activation of endogenous AVP was ablated after intracerebroventricularly administered nesfatin-1/NucB2-neutralizing antibody in comparison with vehicle, without any alteration of water intake nor circadian rhythmicity. These results suggest that chemogenetic activation of endogenous AVP affects, at least in part, central nesfatin-1/NucB2 neurons and may exert anorexigenic effects in the transgenic rats.

**Supplementary Information:**

The online version contains supplementary material available at 10.1186/s12576-021-00802-4.

## Highlights for review

- Chemogenetic activation of AVP suppressed food and water intake.
- Chemogenetic activation of AVP altered circadian rhythmicity.
- AVP activated nesfatin-1 neurons in the CNS.
- Nesfatin-1-neutralizing antibody i.c.v. reversed suppressed food intake.
- Nesfatin-1-neutralizing antibody i.c.v. did not alter circadian rhythmicity.

## Background

The progress of the novel technologies, such as optogenetics and chemogenetics, has been utilized widely. These techniques enable us to investigate the neuron-specific function since we can specifically manipulate the neurons [1, 2]. We have previously generated a transgenic rat line that expressed human muscarinic receptor (hM3Dq) and mCherry exclusively in arginine vasopressin (AVP) neurons [3]. Using the transgenic rats, we have demonstrated that the activation of endogenous AVP suppressed food intake, although the detailed mechanism has not been clarified.

Nesfatin-1/NucB2, identified as a satiety molecule derived from nucleobindin-2 (NucB2) [4], is widely distributed in the central nervous system (CNS) [5]. Nesfatin-1/NucB2 regulates oxytocinergic signaling through a leptin-independent pathway [6]. In addition, nesfatin-1/NucB2-immunoreactive (-ir) neurons were co-localized with AVP-ir neurons in the supraoptic (SON) and paraventricular nuclei (PVN) [7]. However, little is known about the functional relationship between AVP and nesfatin-1/NucB2.

Since both these two molecules (AVP and nesfatin-1/NucB2) are co-expressed in the SON and PVN, and exert anorexigenic effects, we speculated that endogenous AVP might be involved in the activation of nesfatin-1/NucB2 in the CNS, resulted in the suppression of food intake. To test this hypothesis, we examined whether the chemogenetic activation of endogenous AVP, by subcutaneous (s.c.) injection of clozapine-*N*-oxide (CNO), affected nesfatin-1/NucB2 in the CNS by fluorescent immunohistochemistry (FIHC) in the transgenic rats. Neuronal activation was determined by the expression of Fos protein, which is generally used as a marker of neuronal activity [8], co-expressed with nesfatin-1/NucB2-ir neurons. For further elucidation of the anorexigenic action of AVP in the CNS, effects of intracerebroventricularly (i.c.v.) administered nesfatin-1/NucB2-neutralizing antibody (-NA) on feeding suppression after chemogenetic activation of AVP neurons was also examined in the present study.

## Materials and methods

### Animals

Adult male AVP-hM3Dq-mCherry transgenic rats (weighing 300–370 g, each) were group-housed (three rats in a cage) and maintained in the temperature-controlled (23–25 °C) conditions under a 12/12-h light/dark cycle (lights on at 07.00 h). All rats had freely access to standard rat chow and tap water. All rats were handled for 5 days and acclimatized with the single-caged circumstance for 3 days before the experiments. All experiments were performed in strict accordance with ethical guidelines on the use and care of laboratory animals issued by the Physiological Society of Japan and were approved by the Ethics Committee of Animal Care and Experimentation of the University of Occupational and Environmental Health (UOEH) (Approval Number: AE16-012).

### Food and water intake after chemogenetic activation of endogenous AVP

Saline (1 mL/kg, Otsuka Pharmaceutical Co. LTD., Tokyo, Japan) (*n* = 14) or CNO (1 mg/mL/kg, dissolved in 0.9% sterile saline) (*n* = 14) was subcutaneously injected at 30 min before the commencement of the dark cycle (18.30 h). The measurements were started at 19.00 h, zeitgeber time (ZT) 12. Food and water intake was measured at ZT 12, 13, 15, 18, 24/0, and 12. The dosage of CNO was determined according to the previous study [3].

### Circadian activity and core body temperature after chemogenetic activation of endogenous AVP

Nanotag^®^ (Kissei Comtec, Matsumoto, Japan), a telemetry probe which enables us to measure both locomotor activity and core body temperature (CBT) at 5 min intervals, was intraperitoneally (i.p.) implanted into the rats by the dorsal approach after being deeply anesthetized with inhalation of Sevoflurane (Pfizer Japan, Tokyo, Japan). They were allowed to recover for 5 days after the surgery.

On the date of experiment, Saline (1 mL/kg) (*n* = 5) or CNO (1 mg/kg/mL) (*n* = 5) was subcutaneously injected at 30 min before the commencement of the dark cycle, at ZT 11.5. The measurements were started at 19.00 h, at ZT 12, continued for 24 h.

### FIHC for Fos and nesfatin-1/NucB2

At 120 min after s.c. injection of Saline (1 mL/kg, *n* = 5) or CNO (1 mg/kg, *n* = 5), rats were deeply anesthetized with three types of mixed anesthetic agents (in combination with 0.3 mg/kg of medetomidine, 4.0 mg/kg of midazolam, and 5.0 mg/kg of butorphanol). The rats were transcardially perfused with 0.1 M phosphate buffer (PB) (pH 7.4) containing heparin (1000 U/L) and a fixative containing 4% paraformaldehyde (PFA) dissolved in 0.1 M PB. Brains were post-fixed with 4% PFA in 0.1 M PB for 48 h at 4 °C. Tissues were then dehydrated for cryoprotection in 30% sucrose in 0.1 M PB for 48 h at 4 °C. 30-µm-thick serial sections were sliced using a microtome (REM-700, Yamato Kohki Industrial co. ltd., Tokyo, Japan).

Sections were incubated for 12 h at 4 °C with primary antibodies solution (guinea pig anti-c-Fos, Synaptic System, Goettingen, Germany, 1:500, diluted in 0.1 M phosphate-buffered saline with 0.3% Triton X-100 (PBST); rabbit anti-NUCB2, Sigma-Aldrich, MO, USA, 1:2,000, diluted in 0.1 M PBST). After washing twice in PBST, floating sections were incubated for 2 h at room temperature with secondary antibodies (Alexa Fluor 488 goat anti-guinea pig IgG, Abcam, Cambridge, UK, 1:1000 diluted in PBST; Alexa Fluor 405 donkey anti-rabbit IgG, Abcam, Cambridge, UK, 1:1000, diluted in 0.1 M PBST). Sections were rinsed twice in PBS, and then mounted on the slide glass and cover-slipped using a vectashield (Vector Laboratories Co. Ltd., CA, USA).

Examined nuclei in the present study were as follows: SON, PVN, arcuate nucleus (ARC), lateral hypothalamic area (LHA), central nucleus of the amygdala (CeA), ventral tegmental area (VTA), dorsal raphe nucleus (DR), locus coeruleus (LC), area postrema (AP), rostral (r) and caudal (c) part of nucleus tractus solitarius (NTS), and rostral ventrolateral medulla (RVLM). DR were divided into four following subregions; ventrolateral “wings” (DRvl), dorsal (DRd), ventral (DRv), and inter-fascicular (DRi) since each subnucleus exerts differential role [9]. These nuclei were determined according to the coordinates that given in the rat brain atlas [10]. Microphotographs were captured with an All-In-One microscope (BZ-X800, KEYENCE Corporation, Osaka, Japan) and a fluorescence microscope (Nikon, Tokyo, Japan) using the appropriate excitation and emission filters, and a LSM880 confocal laser microscope (Carl Zeiss Co. Ltd. Oberkochen, Germany). Captured images were printed onto a paper in expanded size. They were then blinded and Fos-ir neurons (appearing as round-shaped nuclei), nesfatin-1/NucB2-ir (appearing as cytoplasmic neurons) and their double-ir neurons were counted by two different researchers to avoid skewing the results. Two cross sections (left and right, four nuclei in total per rat per each region) were manually counted and the results were averaged. To prevent double-counting, we checked the cross marks in the printed paper every time we counted the number of Fos-ir, nesfatin-1/NucB2-ir, and double-ir neurons.

### Intracerebroventricular injection of nesfatin-1/NucB2-neutralizing antibody (-NA)

To examine whether the suppressed food intake induced by CNO was due to the activation of nesfatin-1/NucB2 neurons, we performed intracerebroventricular administration of nesfatin-1/NucB2-NA to block the effects of nesfatin-1/NucB2 after s.c. injection of CNO.

Rats were implanted with stainless-steel cannulae aimed at the lateral ventricle. They were deeply anesthetized with three types of mixed anesthetic agents (in combination with 0.3 mg/kg of medetomidine, 4.0 mg/kg of midazolam, and 5.0 mg/kg of butorphanol) and then placed in a stereotaxic frame. A stainless-steel guide cannulae (550 μm outer diameter, 10 mm length) was stereotaxically implanted at the following coordinates: 0.8 mm posterior to the bregma, 1.4 mm lateral to the midline, and 3.0 mm below the surface of the left cortex such that the tip of the cannulae was 1.0 mm above the left cerebral ventricle. Two stainless-steel anchoring screws were fixed to the skull, and the cannulae were secured in place by acrylic dental cement. After the surgical procedure, the animals were handled for 5 days, individually housed in plastic cages, and allowed to recover for 7 days.

Just after s.c. injection of Saline (1 mL/kg) or CNO (1 mg/mL/kg), a stainless-steel injector (300 μm outer diameter) was introduced through the cannulae at a depth of 1.0 mm beyond the end of the guide for intracerebroventricular injection of Vehicle (rabbit IgG, Sigma-Aldrich, MO, USA) or nesfatin-1/NucB2-NA (kindly provided from Dr. Mori, produced in rabbit) at 30 min before the commencement of dark cycle, at ZT 11.5. The total volume of the injected solution of Vehicle (8 µg/4 µL) or nesfatin-1/NucB2-NA (8 µg/4 µL) into the lateral ventricle was 4 μL. The dosage of nesfatin-1/NucB2-NA used in the present study was determined according to the previous studies [4, 11]. All rats had free access to food and water during the measurements. Measurements were conducted until 24 h after the commencement (Saline s.c. + Vehicle i.c.v., *n* = 7; Saline s.c. + nesfatin-1/NucB2-NA i.c.v., *n* = 8; CNO s.c. + Vehicle i.c.v., *n* = 8; CNO s.c. + nesfatin-1/NucB2-NA i.c.v., *n* = 10).

### Statistical analysis

The mean ± SEM was calculated from the results. All data were analyzed by one-way ANOVA followed by a Bonferroni-type adjustment for multiple comparisons (JMP^®^ 15, SAS Institute Inc., Cary, NC, USA). Statistical significance was set at *P* < 0.05.

## Results

### Chemogenetic activation of endogenous AVP suppressed food and water intake with aberrant circadian rhythmicity

**Fig. 1:**
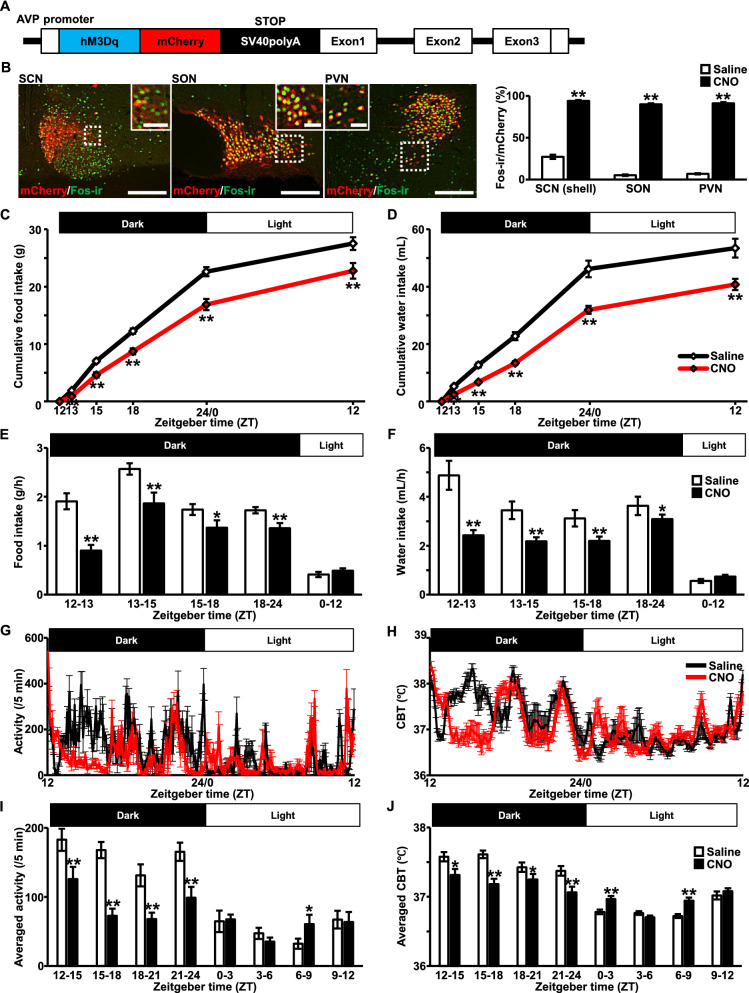
Chemogenetic activation of endogenous arginine vasopressin (AVP) suppressed food and water intake with aberrant circadian rhythmicity. **A** Schematic illustration of a chimeric AVP-hM3Dq-mCherry BAC clone transgene construct. **B** Fos-immunoreactivity (-ir) (green) in mCherry neurons (red) at 120 min after subcutaneous (s.c.) injection of Saline (1 mL/kg) or clozapine-*N*-oxide (CNO, 1 mg/mL/kg) in the suprachiasmatic nucleus (SCN), supraoptic nucleus (SON), and paraventricular nucleus (PVN). The digital images were obtained from the rats treated with CNO. Rectangles encompassed by white dotted lines are magnified in each image. The graph indicates the percentage of Fos expression in mCherry neurons. Scale bars, 200 μm. Scale bars in magnified images, 50 μm. Data are presented as mean ± SEM (*n* = 6, each). **P < 0.01 vs. Saline. Graphs of cumulative food (**C**) and water intake (**D**) after s.c. injection of Saline or CNO are represented. Saline or CNO was subcutaneously administered at 30 min before the commencement of the dark cycle, at ZT 11.5. Food (**E**) and water intake per hour (**F**) are also represented beneath each graph. Data are presented as mean ± SEM (*n* = 14, each). *P < 0.05, **P < 0.01 vs. Saline. Locomotor activity (**G**) and core body temperature (CBT) (**H**) were measured by intraperitoneally implanted telemetry probe (Nanotag^®^). Saline or CNO was subcutaneously administered at 30 min before the commencement of the dark cycle, at ZT 11.5. Averaged activity (per 5 min) (**I**) and averaged CBT (**J**) were calculated every 3 h’ division. Data are presented as mean ± SEM (*n* = 5, each). *P < 0.05, **P < 0.01 vs. Saline

Schematic illustration of a chimeric AVP-hM3Dq-mCherry BAC clone transgene construct is shown in a figure (Fig. 1A). An hM3Dq-mCherry is specifically expressed under the AVP promoter in the transgenic rat brain [3]. Indeed, robust Fos induction was observed at 120 min after s.c. injection of CNO, compared to Saline, exclusively in mCherry-positive AVP neurons of the SCN, SON, and PVN (Fig. 1B), suggesting that an hM3Dq in the transgenic rat line is functioning. All the raw data of the results are represented in the table (Additional file 3: Table S1).

Cumulative food intake was significantly suppressed at ZT 13, 15, 18, 24/0, and 12 after s.c. injection of CNO in comparison to Saline (Fig. 1C). Food intake per unit time (g/h) was calculated from the results at each ZT unit. The analysis revealed that food intake per unit time was significantly suppressed during dark cycle in the rats treated with CNO compared to Saline (Fig. 1E). Cumulative water intake was also significantly suppressed at ZT 13, 15, 18, 24/0, and 12 after s.c. injection of CNO in comparison to Saline (Fig. 1D). Water intake per unit time (mL/h) was calculated from the results at each ZT unit. Water intake per unit time, as well as food intake, was significantly suppressed during dark cycle in the rats treated with CNO compared to Saline (Fig. 1F).

Circadian locomotor activity and CBT were significantly disrupted after CNO injection (Fig. 1G, H). Their averaged per unit time (every 3 h) was calculated from the results. Locomotor activity per unit time was significantly decreased during dark cycle and aberrant behavior was observed during light cycle in the rats treated with CNO compared to Saline (Fig. 1I). Same as locomotor activity, aberrant circadian CBT pattern, lower CBT in dark cycle and higher CBT in light cycle, was observed after s.c. injection of CNO (Fig. 1J). These results were consistent with the previous study [3]. In addition, we confirmed that CNO, at a dosage used in the present study, did not affect food and water intake, nor circadian rhythmicity in adult male wild type rats (Additional file 1: Figure S1).

### Confocal images of Fos expression in nesfatin-1/NucB2-ir neurons in the supraoptic nucleus (SON), paraventricular nucleus (PVN), and arcuate nucleus (ARC) after chemogenetic activation of endogenous AVP

**Fig. 2:**
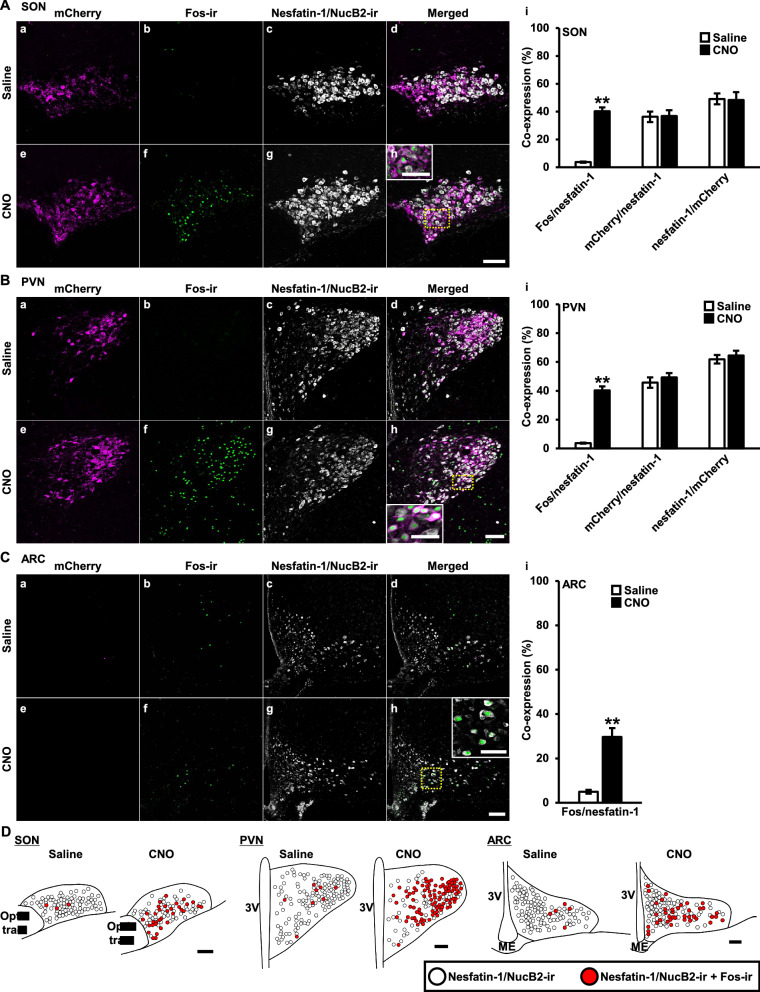
Confocal images of Fos expression in nesfatin-1/NucB2-ir neurons in the supraoptic nucleus (SON), paraventricular nucleus (PVN), and arcuate nucleus (ARC) after chemogenetic activation of endogenous AVP. Confocal images were obtained from the transgenic rats at 120 min after subcutaneously treated with Saline (1 mL/kg) (a–d) or CNO (1 mg/mL/kg) (e–h). mCherry (a, e) (magenta), Fos-ir (b, f) (green), nesfatin-1/NucB2-ir (c, g) (white), and their merged images (d, h) of the SON (**A**), PVN (**B**), and ARC (**C**) are represented. Rectangles encompassed by yellow dotted lines are magnified in the panels (h) (**A**–**C**). Co-expression of the neurons that expressed endogenous fluorescence or immunoreactivity (i) were counted in each nucleus (**A**–**C**). Scale bars, 200 μm. Scale bars in magnified images, 50 μm. Data are presented as mean ± SEM (*n* = 5, each). **P < 0.01 vs. Saline. **D** Nesfatin-1/NucB2-ir, and nesfatin-1/NucB2-ir neurons expressing Fos in the SON, PVN, and ARC either treated with Saline or CNO are mapped by white and red circles, respectively. Scale bars indicate 200 µm. *3V* third ventricle, *ME* median eminence

Confocal laser microscopic observation revealed that Fos immunoreactivity was observed as round-shaped nuclei, whereas, nesfatin-1/NucB2 immunoreactivity was observed as cytoplasmic precipitates. Endogenous mCherry, which was tagged with hM3Dq exclusively in AVP neurons, were seen both in the membrane and cytoplasm of an AVP neurons. Confocal images of the SON (Fig. 2Aa–Ah), PVN (Fig. 2Ba–Bh) and ARC (Fig. 2Ca–Ch) are represented, respectively. Percentage of co-expression in each nucleus are shown in the right side of each nucleus’ confocal images (Fig. 2Ai, Bi, Ci).

Percentages of Fos expression in nesfatin-1/NucB2-ir neurons were dramatically increased in the SON, PVN, and ARC at 120 min after s.c. injection of CNO in comparison to Saline. Consistent with the previous report [7], substantial amount of nesfatin-1/NucB2-ir neurons were co-localized with AVP neurons that were tagged with mCherry in the SON and PVN, and vice versa. Percentages of co-localization were comparable between Saline- and CNO-treated group (Fig. 2Ai, Bi). As AVP neurons are not expressed in the ARC, mCherry was not observed in the ARC (Fig. 2Ca, Ce).

For better interpretation of the results, functional mapping images of the SON, PVN, and ARC, either treated with Saline or CNO, are also represented, respectively (Fig. 2D).

### Functional mapping of Fos expression in nesfatin-1/NucB2-ir neurons after chemogenetic activation of endogenous AVP

**Fig. 3:**
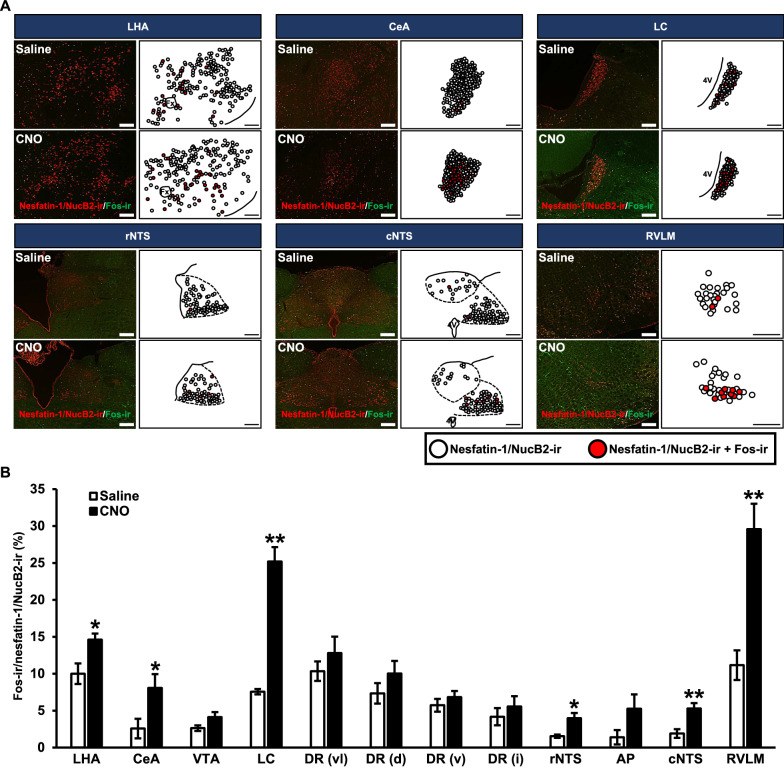
Functional mapping of Fos expression in nesfatin-1/NucB2-ir neurons after chemogenetic activation of endogenous AVP. **A** Digital images of fluorescent immunohistochemistry for nesfatin-1/NucB2 (red) and Fos (green) in the lateral hypothalamic area (LHA), central nucleus of the amygdala (CeA), locus coeruleus (LC), rostral part of nucleus tructus solitalis (rNTS), caudal part of nucleus tructus solitalis (cNTS), and rostral ventrolateral medulla (RVLM). Each schematic illustration indicates nesfatin-1/NucB2-ir neurons (white circle) and co-localization of nesfatin-1/NcB2 neurons expressing Fos (red circle) in each nucleus. The images were obtained from the rats at 120 min after s.c. injection of Saline or CNO. The scale bars indicate 200 µm. Fx, fornix; 4V, fourth ventricle. **B** The percentages of nesfatin-1/NucB2-ir neurons expressing Fos-ir at 120 min after s.c. injection of CNO. Data are presented as means ± SEM (*n* = 5, each). *P < 0.05, **P < 0.01 vs. Saline-treated group. *VTA* ventral tegmental area, *DR* dorsal raphe nucleus, *AP* area postrema. DR were divided into four following subregions; dorsal (DRd), ventral (DRv), ventrolateral “wings” (DRvl), and inter-fascicular (DRi)

Digital images and schematic illustration of the examined nuclei of Fos-ir in nesfatin-1/NucB2-ir neurons are represented (Fig. 3A). In digital images, nesfatin-1/NucB2-ir neurons were identified as red cytoplasmic precipitates and Fos-ir expressing neurons were identified as green nuclei. Representative functional mapping images of nesfatin-1/NucB2-ir neurons and co-localization of nesfatin-1/NucB2-ir neurons expressing Fos were demonstrated, indicated by white and red circle, respectively. The nesfatin-1/NucB2 expressed nuclei that were altered by s.c. injection of CNO were delineated in the figure. Greater number of nesfatin-1/NucB2-ir with Fos-ir was observed following s.c. injection of CNO in the LHA, CeA, LC, rNTS, cNTS, and RVLM (Fig. 3A).

The percentage of nesfatin-1/NucB2-ir neurons were comparable in the VTA, DRvl, DRd, DRv, DRi, and AP (Fig. 3B). They were significantly increased after s.c. injection of CNO compared to Saline in the LHA, CeA, LC, rNTS, cNTS, and RVLM (Fig. 3B).

We also carried out FIHC for AVP and nesfatin-1/NucB2 to recognize the axons of AVP neurons and/or nesfatin-1/NucB2 positive AVP neurons (Additional file 2: Figure S2). AVP-ir and/or nesfatin-1/NucB2-ir positive AVP axons were observed in the brain regions where Fos induction in nesfatin-1/NucB2-ir neurons was observed after chemogenetic activation of AVP. Some of the axons were immunoreactive with AVP alone. These results indicated that AVP neurons as well as nesfatin-1/NucB2-ir positive AVP-ir neurons affected the nesfatin-1/NucB2-ir neurons in the brain regions other than the SON and PVN, although the origin (i.e., SON or PVN) of the axons could not be specified by means of this method.

### Suppressed food intake induced by chemogenetic activation of AVP neurons was attenuated after i.c.v. administered nesfatin-1/NucB2-neutralizing antibody (-NA) without any alteration of water intake nor circadian rhythmicity

**Fig. 4:**
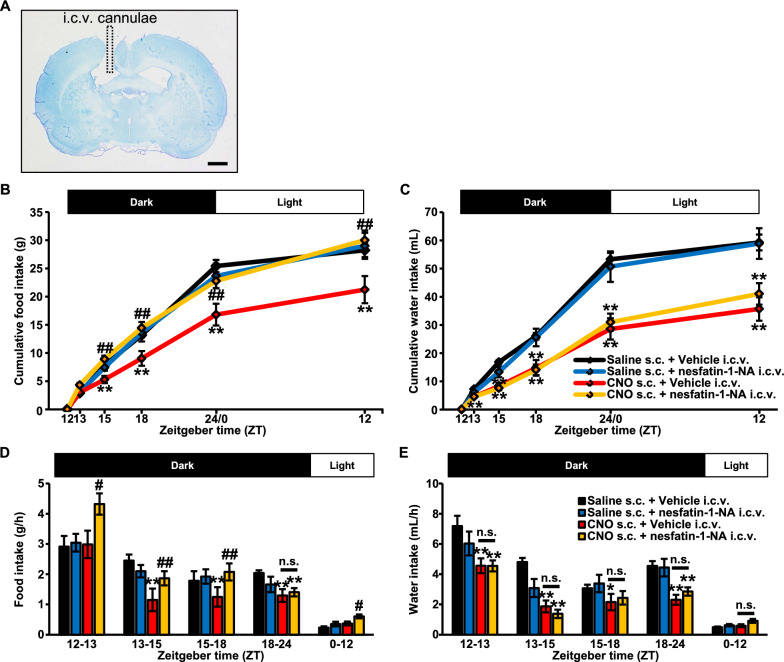
Suppressed food intake induced by chemogenetic activation of AVP neurons was attenuated after i.c.v. administered nesfatin-1/NucB2-neutralizing antibody (-NA) without any alteration of water intake. **A** Digital image of an implanted stainless-steel cannulae aimed at the lateral ventricle. Vehicle (8 µg/4 µL) or Nesfatin-1/NucB2-neutralizing antibody (-NA) (8 µg/4 µL) was i.c.v. administered at 30 min prior to the start of the dark cycle, at ZT 11.5. Just after the intracerebroventricular injection, Saline or CNO (1 mg/mL/kg) was subcutaneously administered. The measurements were commenced at ZT 0. Cumulative food (g) (**B**) and water intake (mL) (**C**), and their intake per unit time (g/h, mL/h) (**D**, **E**) were calculated from the results. All rats had free access to food and water during the measurements. Data are presented as means ± SEM (Saline s.c. + Vehicle i.c.v., *n* = 7; Saline s.c. + nesfatin-1/NucB2-NA i.c.v., *n* = 8; CNO s.c. + Vehicle i.c.v., *n* = 8; CNO s.c. + nesfatin-1/NucB2-NA i.c.v., *n* = 10). *P < 0.05, **P < 0.01 vs. Saline s.c. + Vehicle i.c.v. ^#^*P* < 0.05, ^##^*P* < 0.01 vs. CNO s.c. + Vehicle i.c.v

A cannulae-implanted-brain slice with toluidine blue staining was represented in a figure (Fig. 4A). An implanted stainless-steel cannulae was aimed at the lateral ventricle. The rats with improperly inserted cannulae were excluded from the analysis.

I.c.v. injected nesfatin-1/NucB2-NA did not affect food intake nor water intake in the rats treated with s.c. injection of Saline (Fig. 4B, C). Same as cumulative food and water intake, their intake per unit time (g/h, mL/h) was not altered in Saline-treated rats (Fig. 4D, E).

In the rats treated with s.c. injection of CNO, however, suppressed food intake was dramatically attenuated by i.c.v. administered nesfatin-1/NucB2-NA (Fig. 4B). Food intake per unit time (g/h) was also significantly increased after intracerebroventricular injection of nesfatin-1/NucB2-NA (Fig. 4D).

Strikingly, suppressed water intake, which was induced by s.c. injection of CNO, was not altered after i.c.v. administered nesfatin-1/NucB2-NA (Fig. 4C). Water intake per unit time was not altered, neither (Fig. 4E).

**Fig. 5:**
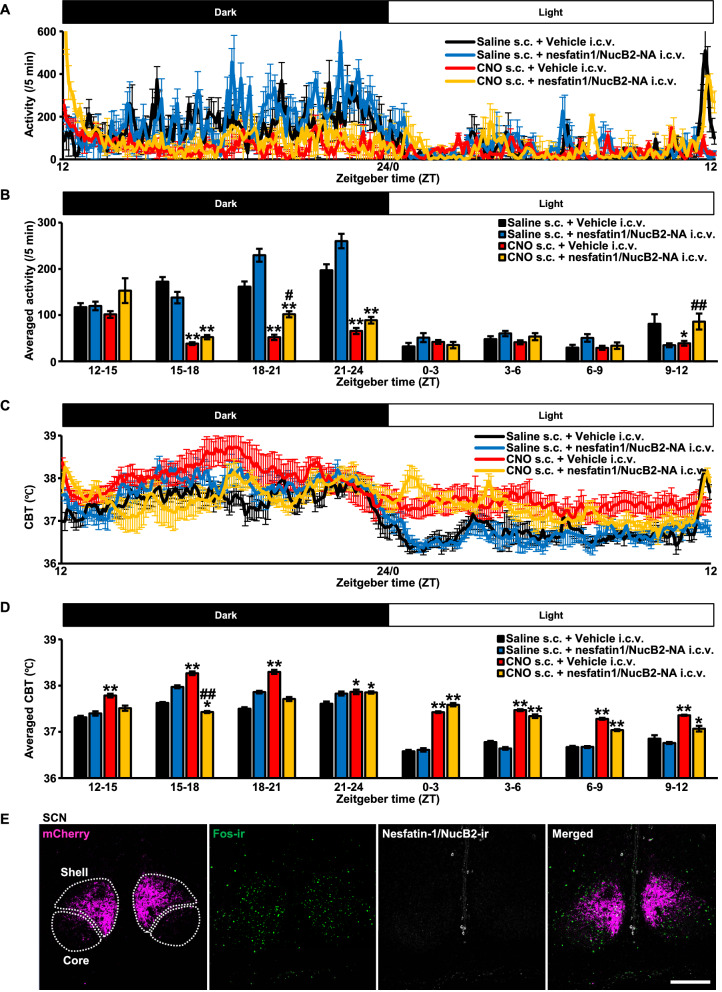
Circadian rhythmicity was not altered by i.c.v. injected nesfatin-1/NucB2-neutralizing antibody (-NA). Locomotor activity (**A**) and core body temperature (CBT) (**C**) were measured by intraperitoneally implanted telemetry probe (Nanotag^®^). Vehicle (8 µg/4 µL) or Nesfatin-1/NucB2-neutralizing antibody (-NA) (8 µg/4 µL) was i.c.v. administered at 30 min prior to the start of the dark cycle, at ZT 11.5. Just after the intracerebroventricular injection, Saline or CNO (1 mg/mL/kg) was subcutaneously (s.c.) administered. The measurements were commenced at ZT 0. Averaged activity (per 5 min) (**B**) and averaged CBT (**D**) were calculated every 3 h division. Data are presented as mean ± SEM (*n* = 5, each). *P < 0.05, **P < 0.01 vs. Saline s.c. + Vehicle i.c.v. ^#^*P* < 0.05, ^##^*P* < 0.01 vs. CNO s.c. + Vehicle i.c.v. **E** Immunohistochemical analysis of nesfatin-1/NucB2 in the suprachiasmatic nucleus (SCN)

To confirm i.c.v. administered nesfatin-1/NucB2-NA did not affect circadian rhythmicity, circadian locomotor activity and CBT were measured by i.p. implanted telemetry probe as described in the previous figure (Fig. 1G–J). Circadian locomotor activity and CBT were significantly disrupted after CNO injection, which was consistent with the previous results (Fig. 1G, H), however, they were not affected by i.c.v. injected nesfatin-1/NucB2-NA (Fig. 5A, C). Analysis revealed that locomotor activity per unit time (every 3 h) was significantly disrupted in the rats treated with CNO compared to Saline, but was not significantly altered by i.c.v. injected nesfatin-1/NucB2-NA (Fig. 5B).

Same as locomotor activity, aberrant circadian CBT pattern was observed after s.c. injection of CNO, but was not altered by i.c.v. injected nesfatin-1/NucB2-NA (Fig. 5D).

Nesfatin-1/NucB2 immunoreactivity was not observed in the SCN of adult male AVP-hM3Dq-mCherry transgenic rats (Fig. 5E). The image was obtained from a rat treated with CNO. These results suggest that even if nesfatin-1/NucB2-ir neurons, which were co-localized with AVP neurons, might be stimulated alongside with chemogenetic activation of AVP in the SON and PVN, nesfatin-1/NucB2 nor nesfatin-1/NucB2-NA did not directly affect their circadian rhythmicity.

## Discussion

The obtained data demonstrates that in our transgenic rats chemogenetic activation of endogenous AVP affected central nesfatin-1/NucB2 pathways and could exert anorexigenic effects in the transgenic rats. Although these data do not define a precise role of AVP in anorexia, we hypothesized that AVP-induced nesfatin-1/NucB2 feeding suppression observed in the present study was affected by two distinct pathways. One is AVP in the SON and PVN. AVP neurons in these nuclei send their axons into various brain region, and may activate nesfatin-1/NucB2 neurons. The other is AVP in the SCN. AVP neurons in the SCN play a pivotal role in maintaining circadian rhythmicity and entrain to the environmental light/dark cycle [12]. Indeed, circadian disruption causes feeding suppression [13]. Circadian AVP synthesis reaches nadir during dark cycle [14]. Chemogenetic activation of AVP was executed at this time point in the present study, which may cause significant disruption of the intrinsic circadian rhythmicity, resulting in feeding suppression.

About 40–60% of AVP-ir neurons were co-localized with nesfatin-1/NucB2-ir neurons in the SON and PVN in the transgenic rats in the present study. Over 90% of AVP neurons were activated after s.c. injection of CNO in the transgenic rats. Taken together, some of the nesfatin-1/NucB2 neurons in the SON and PVN were activated by chemogenetic activation of AVP.

Nesfatin-1/NucB2 neurons activate oxytocin neurons in the PVN and exert anorexigenic effects through pro-opiomelanocortin neurons in the NTS [6]. Price et al. have reported that nesfatin-1/NucB2 inhibits NPY neurons in the ARC [15]. It is, thus, compatible that activated nesfatin-1/NucB2 neurons in the PVN and ARC induced feeding suppression.

CeA is one of the important nuclei that are involved in feeding control [16]. It has been suggested that nesfatin-1/NucB2 in the amygdala plays an important role in decreasing gastric motility [17]. Nesfatin-1/NucB2 neurons in the NTS are sensitive to gastric distension and may contribute to the satiety signal [18]. Similarly, nesfatin-1/NucB2 signaling in the LHA participates in the regulation of efferent information to the peripheral gastrointestinal tract and gastric secretion [19]. Since increased number of nesfatin-1/NucB2-ir neurons was observed in the CeA, NTS, and LHA after chemogenetic activation, AVP may exert anorexigenic effects via CeA-, NTS-, and LHA-nesfatin-1/NucB2 pathway.

Nesfatin-1/NucB2 immunoreactivity is co-expressed with neuropeptide Y and cocaine- and amphetamine-regulated transcript (CART) in the LC and DR in human [20]. Noradrenalin and serotonin (5-HT) producing neurons are localized in the LC and DR, respectively, and these monoamines are involved in wide variety of neuronal activity, including feeding. RVLM is one of the nuclei that controls sympathetic nervous system. RVLM receives neuronal projections from the hypothalamus [21]. It is speculated that chemogenetic activation of AVP affected nesfatin-1/NucB2 neurons in the LC and RVLM, then caused feeding suppression via sympathetic nervous system. The autonomic nervous system consists of sympathetic and parasympathetic nerve. Generally, the activation of the autonomic nervous system is contradictory, when one is activated, the other is inhibited [22]. Orexigenic neuropeptides, such as Neuropeptide Y, orexin, and galanin increased food intake and decreased sympathetic nerve activity. In contrast, anorexigenic neuropeptides, such as cholecystokinin (CCK-8), corticotrophin-releasing hormone (CRH), leptin and CART suppressed food intake and increased sympathetic nerve activity [23]. Many studies have revealed that the inhibition of parasympathetic nerve decreases gastrointestinal motility, resulting in anorexia [24, 25]. On the other hand, NamKoong et al. have reported that direct activation of parasympathetic nerves suppressed food intake [26]. Thus, from the present results, the cause-and-effect relationship between feeding suppression and autonomic nervous system remains unclear, which should be clarified by further study.

We utilized c-Fos as an indicator of neuronal activation [8]. Of note, we could detect only activated neurons, not those potentially inhibited. It is possible that feeding suppression may be achieved by inhibiting orexigenic neuropeptides.

Nesfatin-1/NucB2 suppressed water intake as well as food intake [27], which was consistent with our results. However, i.c.v. injected nesfatin-1/NucB2-NA did not ablate suppressed water intake that was induced by chemogenetic activation of AVP. The effect of AVP on water intake may be much robust than that of nesfatin-1/NucB2. In addition, i.c.v. injected nesfatin-1/NucB2-NA did not affect circadian rhythmicity. These results were reasonable because, even if the food intake was suppressed by circadian disruption or AVP per se, the chemogenetic activation of AVP eventually caused an activation of nesfatin-1/NucB2 neurons, resulting in feeding suppression.

It remains unclear whether AVP or nesfatin-1/NucB2 in the SON and PVN affect nesfatin-1/NucB2 neurons in the nuclei other than the SON and PVN since chemogenetic activation may activate both AVP and nesfatin-1/NucB2 neurons in the SON and PVN. In addition, i.c.v. injected nesfatin-1/NucB2-NA canceled AVP-induced anorexigenic effects. It is possible that nesfatin-1/NucB2, but not AVP release from the AVP neurons, mediated the anorexigenic function of AVP neurons. The other reasonable explanation is that AVP was also involved in the anorexigenic action but nesfatin-1/NucB2 in the downstream was critical. Pre-treatment with AVP receptor antagonist, such as V1a and V1b antagonist, would elucidate further mechanism of AVP on nesfatin-1/NucB2-induced feeding suppression.

## Conclusions

In conclusion, we have demonstrated that chemogenetic activation of endogenous AVP affected nesfatin-1/NucB2 pathways in the transgenic rats. Besides its effect on feeding, AVP also has crucial roles in metabolism [28]. Increasing tendencies of overeating highly palatable food is most likely a contributing factor in the WHO 2021 World Health Organization’s reported current obesity epidemic, with the associated increase in incidence of metabolic syndrome and type 2 diabetes. Since AVP is intrinsically regulates feeding behavior, further clarification of the underlying mechanisms of AVP on nesfatin-1/NucB2 pathway would contribute to the identification of potential therapeutic targets for the treatment of metabolic disorders in the future.

### Supplementary Information

**Additional file 1: Figure S1.** Subcutaneously injected clozapine-N-oxide (CNO, 1 mg/kg) did not alter food and water intake nor circadian activity and core body temperature (CBT) in adult male Wild Type (WT) rats. Saline (1 mL/kg, n=8) or CNO (1 mg/kg, n=8) was subcutaneously injected to adult male WT rats (300-350 g) at 30 min prior to the commencement of a dark period, ZT 11.5. Food and water intake was not altered after CNO administration. Circadian activity and CBT (n=5, each) were not disrupted by CNO in WT rats, suggesting that CNO did not affect these behaviors. Data are presented as mean ± SEM.

**Additional file 2: Figure S2.** AVP-immunoreactive (-ir) and nesfatin-1/nNucB2-ir neurons in the SON and PVN send their dendrites into various brain regions. Fluorescent immunohistochemistry (FIHC) for AVP and nesfatin-1/NucB2 was performed to reveal the neuronal connectivity between the SON/PVN and other brain regions. Dendrites were confirmed in the slice of the hypothalamus (A). AVP-ir and/or AVP/nesfatin-1/NucB2-ir dendrites were observed in the LHA (B), ARC (C), CeA (D), LC (E), and NTS (F), where Fos induction in nesfatin-1/NucB2-ir neurons was observed after chemogenetic activation of AVP. Some of the dendrites were immunoreactive with AVP alone. Scale bars indicate 200 μm. Red, green, and yellow arrow heads indicate the dendrite that was immunoreactive with AVP (red), nesfatin-1/NucB2 (green), and both of them (yellow), respectively. Abbreviations: supraoptic nucleus, SON; paraventricular nucleus, PVN; lateral hypothalamic area, LHA; central nucleus of the amygdala, CeA; locus coeruleus, LC; nucleus tractus solitarius, NTS.

**Additional file 3: Table S1.** Raw data of the results.

## Data Availability

The datasets used and analyzed during the current study are available from the corresponding authors on reasonable request.

## Abbreviations

AP: Area postrema
ARC: Arcuate nucleus
AVP: Arginine vasopressin
CART: Cocaine- and amphetamine-regulated transcript
CBT: Core body temperature
CCK-8: Cholecystokinin-8
CeA: Central nucleus of the amygdala
CNO: Clozapine-*N*-oxide
CNS: Central nervous system
cNTS: Caudal part of nucleus tractus solitarius
CRH: Corticotrophin-releasing hormone
DR: Dorsal raphe nucleus
DRd: Dorsal part of dorsal raphe
DRi: Inter-fascicular part of dorsal raphe
DRv: Ventral part of dorsal raphe
DRvl: Ventrolateral “wings” part of dorsal raphe
FIHC: Fluorescent immunohistochemistry
hM3Dq: Human M3 muscarinic cholinergic Gq-coupled receptor
i.c.v.: Intracerebroventricularly
i.p.: Intraperitoneally
-ir: -Immunoreactive
LC: Locus coeruleus
LHA: Lateral hypothalamic area
-NA: -Neutralizing antibody
PB: Phosphate buffer
PBST: Phosphate-buffered saline with 0.3% Triton X-100
PFA: Paraformaldehyde
PVN: Paraventricular nucleus
rNTS: Rostral part of nucleus tractus solitarius
RVLM: Rostral ventrolateral medulla
s.c.: Subcutaneous
SON: Supraoptic nucleus
VTA: Ventral tegmental area
ZT: Zeitgeber time
