# Supplementary material for: Chemogenetic activation of endogenous arginine vasopressin exerts anorexigenic effects via central nesfatin-1/NucB2 pathway
Source: J Physiol Sci. 2021 Jun 16;71:18. doi: 10.1186/s12576-021-00802-4 (PMC10717637; doi:10.1186/s12576-021-00802-4)
Supplement: Supplementary file 1 — Additional file 1: Figure S1. Subcutaneously injected clozapine-N-oxide (CNO, 1 mg/kg) did not alter food and water intake nor circadian activity and core body temperature (CBT) in adult male Wild Type (WT) rats. Saline (1 mL/kg, n=8) or CNO (1 mg/kg, n=8) was subcutaneously injected to adult male WT rats (300-350 g) at 30 min prior to the commencement of a dark period, ZT 11.5. Food and water intake was not altered after CNO administration. Circadian activity and CBT (n=5, each) were not disrupted by CNO in WT rats, suggesting that CNO did not affect these behaviors. Data are presented as mean ± SEM. [file 12576_2021_802_MOESM1_ESM.pptx]

## Slide 1
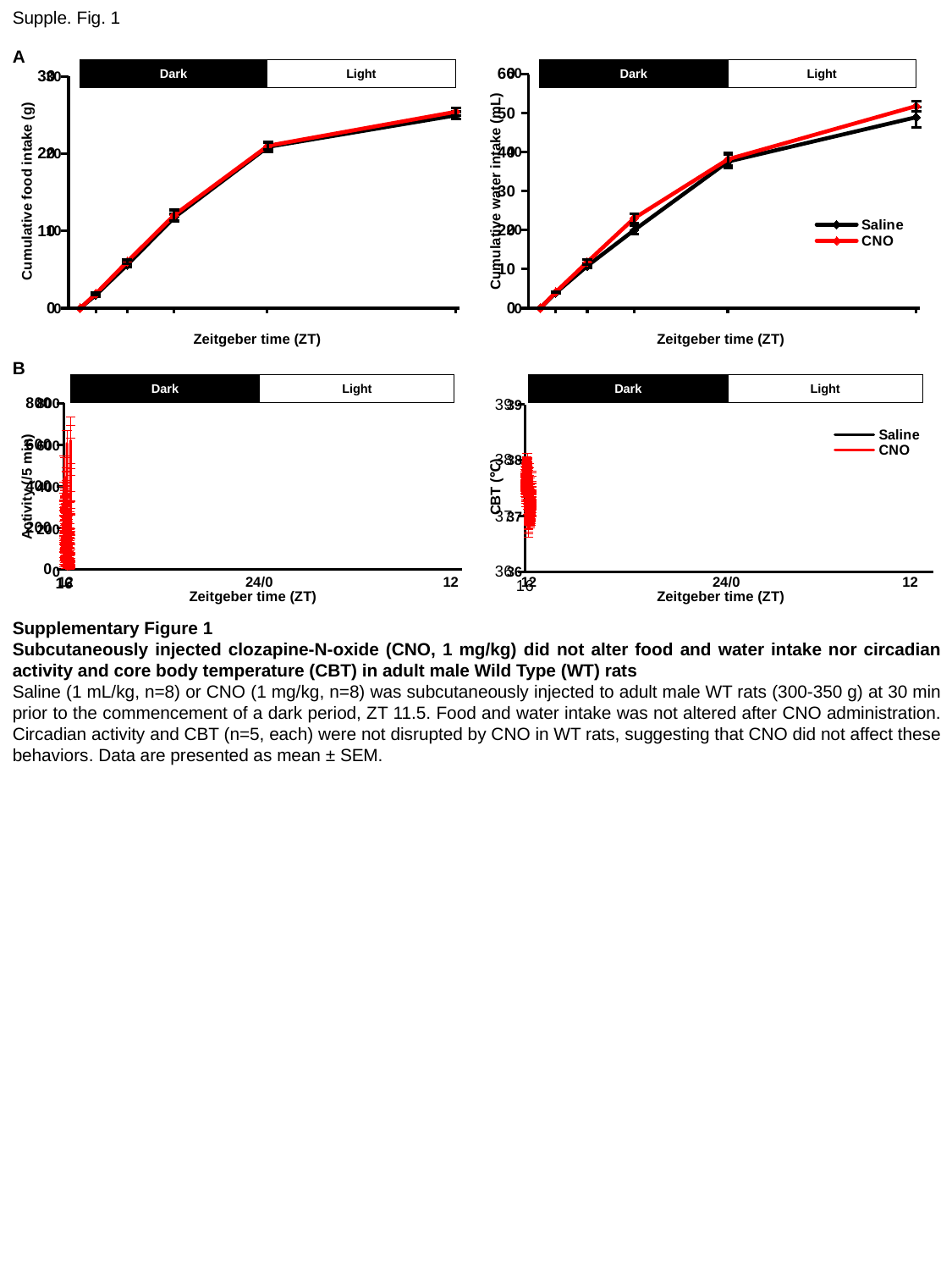

Supple. Fig. 1
A
60
Dark
Light
Dark
Light
30
### Chart
| Category | Saline | CNO |
|---|---|---|
| | None | None |
| 12 | 0.0 | 0.0 |
| | None | None |
| 13 | 1.7000000000000002 | 1.875 |
| | None | None |
| | None | None |
| | None | None |
| 15 | 5.6 | 6.0125 |
| | None | None |
| | None | None |
| | None | None |
| | None | None |
| | None | None |
| 18 | 11.7375 | 12.049999999999999 |
| | None | None |
| | None | None |
| | None | None |
| | None | None |
| | None | None |
| | None | None |
| | None | None |
| | None | None |
| | None | None |
| | None | None |
| | None | None |
| 24/0 | 20.900000000000002 | 21.037499999999998 |
| | None | None |
| | None | None |
| | None | None |
| | None | None |
| | None | None |
| | None | None |
| | None | None |
| | None | None |
| | None | None |
| | None | None |
| | None | None |
| | None | None |
| | None | None |
| | None | None |
| | None | None |
| | None | None |
| | None | None |
| | None | None |
| | None | None |
| | None | None |
| | None | None |
| | None | None |
| | None | None |
| 12 | 24.975 | 25.4375 |
### Chart
| Category | Saline | CNO |
|---|---|---|
| | None | None |
| 12 | 0.0 | 0.0 |
| | None | None |
| 13 | 3.9374999999999996 | 4.1499999999999995 |
| | None | None |
| | None | None |
| | None | None |
| 15 | 10.787499999999998 | 11.812499999999998 |
| | None | None |
| | None | None |
| | None | None |
| | None | None |
| | None | None |
| 18 | 20.025000000000002 | 23.062500000000004 |
| | None | None |
| | None | None |
| | None | None |
| | None | None |
| | None | None |
| | None | None |
| | None | None |
| | None | None |
| | None | None |
| | None | None |
| | None | None |
| 24/0 | 37.6 | 38.18749999999999 |
| | None | None |
| | None | None |
| | None | None |
| | None | None |
| | None | None |
| | None | None |
| | None | None |
| | None | None |
| | None | None |
| | None | None |
| | None | None |
| | None | None |
| | None | None |
| | None | None |
| | None | None |
| | None | None |
| | None | None |
| | None | None |
| | None | None |
| | None | None |
| | None | None |
| | None | None |
| | None | None |
| 12 | 48.9625 | 51.81249999999999 |40
20
Cumulative food intake (g)
Cumulative water intake (mL)
20
10
0
0
Zeitgeber time (ZT)
Zeitgeber time (ZT)
B
Dark
Light
Dark
Light
800
39
### Chart
| Category | Saline | CNO |
|---|---|---|
### Chart
| Category | Saline | CNO |
|---|---|---|600
38
Activity (/5 min)
CBT (℃)
400
37
200
36
0
12
24/0
12
12
24/0
12
Zeitgeber time (ZT)
Zeitgeber time (ZT)
Supplementary Figure 1
Subcutaneously injected clozapine-N-oxide (CNO, 1 mg/kg) did not alter food and water intake nor circadian activity and core body temperature (CBT) in adult male Wild Type (WT) rats
Saline (1 mL/kg, n=8) or CNO (1 mg/kg, n=8) was subcutaneously injected to adult male WT rats (300-350 g) at 30 min prior to the commencement of a dark period, ZT 11.5. Food and water intake was not altered after CNO administration. Circadian activity and CBT (n=5, each) were not disrupted by CNO in WT rats, suggesting that CNO did not affect these behaviors. Data are presented as mean ± SEM.
