# Supplementary material for: Chemogenetic activation of endogenous arginine vasopressin exerts anorexigenic effects via central nesfatin-1/NucB2 pathway
Source: J Physiol Sci. 2021 Jun 16;71:18. doi: 10.1186/s12576-021-00802-4 (PMC10717637; doi:10.1186/s12576-021-00802-4)
Supplement: Supplementary file 2 — Additional file 2: Figure S2. AVP-immunoreactive (-ir) and nesfatin-1/nNucB2-ir neurons in the SON and PVN send their dendrites into various brain regions. Fluorescent immunohistochemistry (FIHC) for AVP and nesfatin-1/NucB2 was performed to reveal the neuronal connectivity between the SON/PVN and other brain regions. Dendrites were confirmed in the slice of the hypothalamus (A). AVP-ir and/or AVP/nesfatin-1/NucB2-ir dendrites were observed in the LHA (B), ARC (C), CeA (D), LC (E), and NTS (F), where Fos induction in nesfatin-1/NucB2-ir neurons was observed after chemogenetic activation of AVP. Some of the dendrites were immunoreactive with AVP alone. Scale bars indicate 200 μm. Red, green, and yellow arrow heads indicate the dendrite that was immunoreactive with AVP (red), nesfatin-1/NucB2 (green), and both of them (yellow), respectively. Abbreviations: supraoptic nucleus, SON; paraventricular nucleus, PVN; lateral hypothalamic area, LHA; central nucleus of the amygdala, CeA; locus coeruleus, LC; nucleus tractus solitarius, NTS. [file 12576_2021_802_MOESM2_ESM.pptx]

## Slide 1
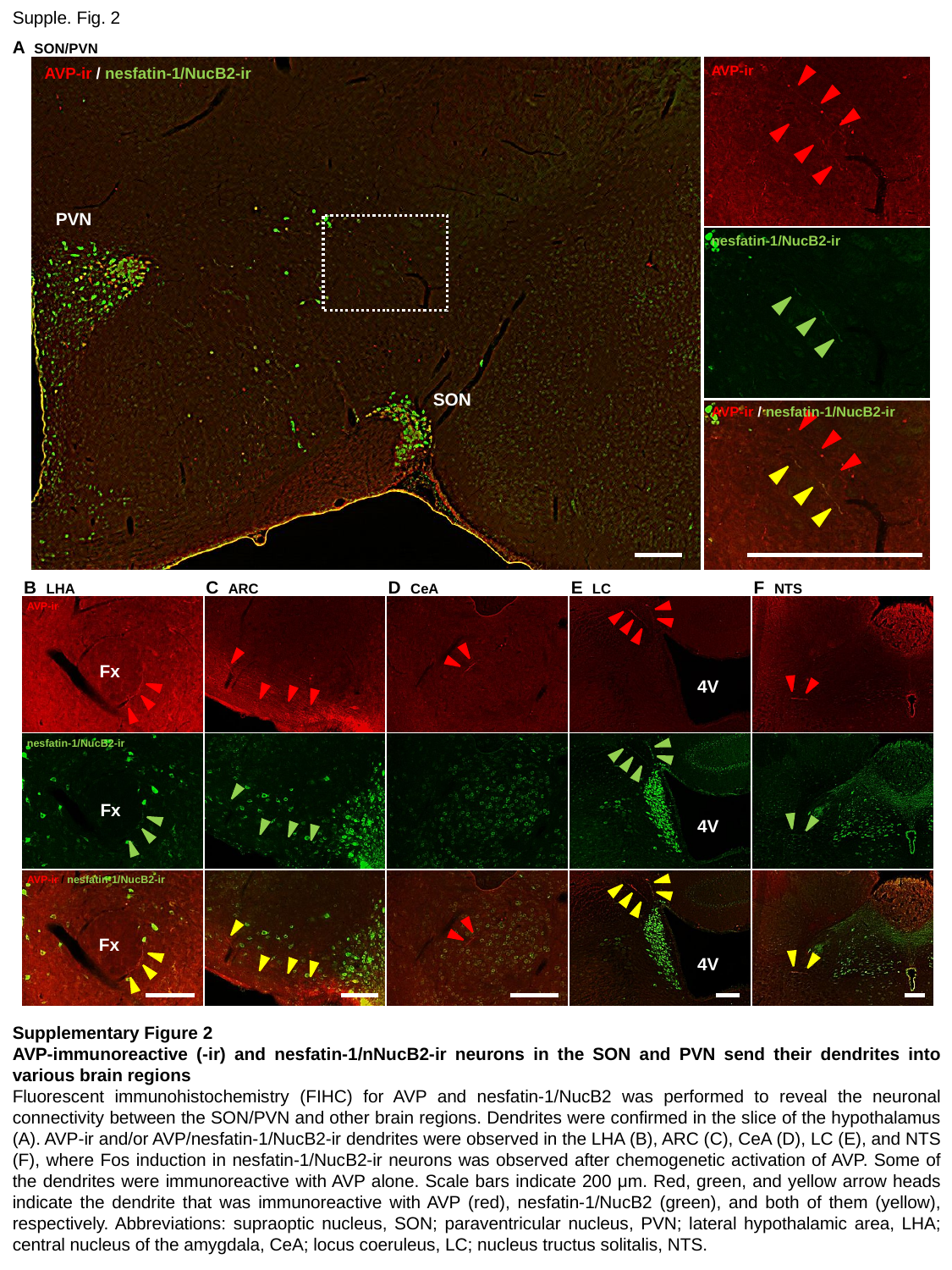

Supple. Fig. 2
A SON/PVN
AVP-ir
AVP-ir / nesfatin-1/NucB2-ir
PVN
SON
nesfatin-1/NucB2-ir
AVP-ir / nesfatin-1/NucB2-ir
B LHA
C ARC
D CeA
E LC
F NTS
AVP-ir
Fx
4V
nesfatin-1/NucB2-ir
Fx
4V
AVP-ir / nesfatin-1/NucB2-ir
Fx
4V
Supplementary Figure 2
AVP-immunoreactive (-ir) and nesfatin-1/nNucB2-ir neurons in the SON and PVN send their dendrites into various brain regions
Fluorescent immunohistochemistry (FIHC) for AVP and nesfatin-1/NucB2 was performed to reveal the neuronal connectivity between the SON/PVN and other brain regions. Dendrites were confirmed in the slice of the hypothalamus (A). AVP-ir and/or AVP/nesfatin-1/NucB2-ir dendrites were observed in the LHA (B), ARC (C), CeA (D), LC (E), and NTS (F), where Fos induction in nesfatin-1/NucB2-ir neurons was observed after chemogenetic activation of AVP. Some of the dendrites were immunoreactive with AVP alone. Scale bars indicate 200 μm. Red, green, and yellow arrow heads indicate the dendrite that was immunoreactive with AVP (red), nesfatin-1/NucB2 (green), and both of them (yellow), respectively. Abbreviations: supraoptic nucleus, SON; paraventricular nucleus, PVN; lateral hypothalamic area, LHA; central nucleus of the amygdala, CeA; locus coeruleus, LC; nucleus tructus solitalis, NTS.
